# Intratracheal administration of siRNA targeting FAS reduces ischemia-reperfusion-induced lung injury

**DOI:** 10.1186/cc10700

**Published:** 2012-03-20

**Authors:** L Del Sorbo, G Muraca, A Costamagna, G Rotondo, L Laudari, F Civiletti, E Tonoli, E Martin, V Fanelli, V Ranieri

**Affiliations:** 1 University of Turin, Italy

## Introduction

Ischemia-reperfusion injury is one of the main causes of primary graft dysfunction after lung transplantation. Fas-mediated apoptosis plays a major role in the pathogenesis of ischemia-reperfusion injury. Exogenous administration of small interfering RNA (siRNA) is an effective strategy to specifically silence the expression of proteins through blocking the translation of mRNA. The aim of this study was to investigate in an *ex vivo *mouse model of lung ventilation and perfusion whether a specific siRNA targeting Fas is able to reduce ischemia-reperfusion injury.

## Methods

C57BL/6 male mice were randomized to intratracheally receive a specific sequence of siRNA targeting FAS (siRNA-FAS) or a scrambled siRNA 48 hours before undergoing 6 hours of cold ischemic time (4°C) followed by 2 hours of *ex vivo *ventilation (peak inspiratory pressure = 7 cmH_2_O, PEEP = 2 cmH_2_O, respiratory rate = 100 breaths/minute, FiO_2 _= 100%) and reperfusion (4% bovine serum albumin RPMI medium with 10% fresh blood at 1 ml/minute flow rate) in a predisposed humidified chamber at 37°C. At the end of the experiment, lung elastance, assessed through tidal volume, and total protein concentration in the bronchoalveolar lavage (BAL) fluid were measured. A separate set of lungs were analysed by western blot before undergoing cold ischemia to assess the expression of FAS protein.

## Results

The intratracheal administration of siRNA-FAS reduced the expression of FAS in the lung by 44% (siRNA-FAS 0.90 ± 0.11 vs. scrambled siRNA 1.61 ± 0.18 AU). Lung elastance and BAL total protein concentration were significantly reduced in the siRNA-FAS group as compared to control in lungs exposed to 6 hours of cold ischemia followed by 2 hours of reperfusion. See Table 1.

**Table 1:** 

	siRNA-FAS	siRNA scrambled
Elastance (cmH_2_O/ml)	11.34 ± 0.24*	13.75 ± 0.99
BAL proteins (μg/ml)	529.1 ± 64.8*	928.5 ± 138.2

Data are mean ± SE. Comparison between groups was performed with the Student's *t *test. *P <0.05.

## Conclusion

The intratracheal administration of siRNA targeting FAS prevents the increase of the alveolar membrane permeability during ischemia-reperfusion injury.

